# Supplementary material for: Targeting EZH2 histone methyltransferase activity alleviates experimental intestinal inflammation
Source: Nat Commun. 2019 Jun 3;10:2427. doi: 10.1038/s41467-019-10176-2 (PMC6547712; doi:10.1038/s41467-019-10176-2)
Supplement: Supplementary file 1 — Supplementary Information [file 41467_2019_10176_MOESM1_ESM.pdf]

Supplementary Information

Targeting EZH2 Histone Methyltransferase Activity Alleviates Experimental Intestinal Inflammation

Zhou et al.

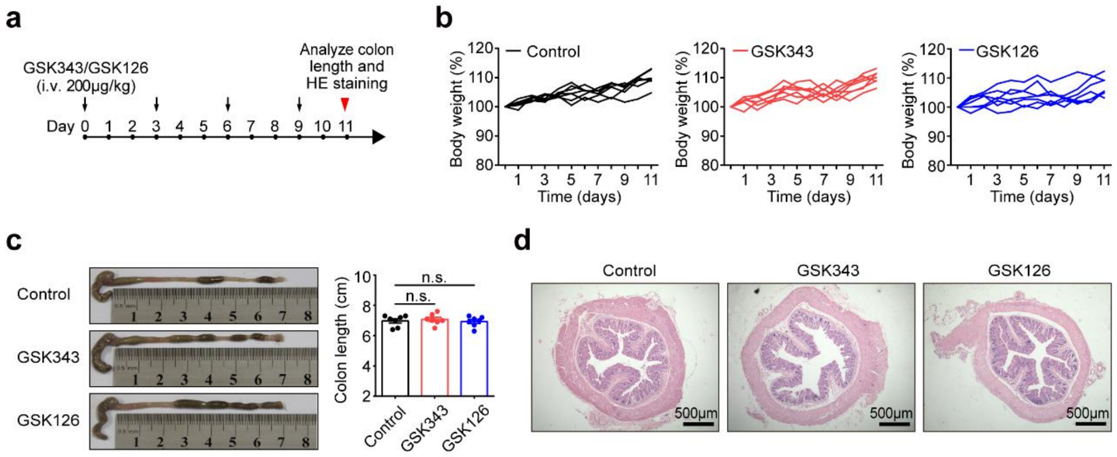

Supplementary Figure 1. GSK343 and GSK126 are safe to mice. (**a**) Protocol for GSK343 and GSK126 treatment in C57BL/6 mice. (**b**) Body weight of mice that injected with vehicle (control) or GSK343 or GSK126 (n=7 per group). (**c, d**) Colon length (**c**) and representative hematoxylin and eosin (H&E) staining of histological sections of the distal colon (**d**) at day 11 (n = 7 per group). Data are representative of three independent experiments. The statistical significance of differences was determined by one-way analysis of variance followed by Bonferroni post-test (**c**). n.s. = not significant. Results are presented as the means ± standard error of the mean (SEM). Source data are provided as a Source Data file.

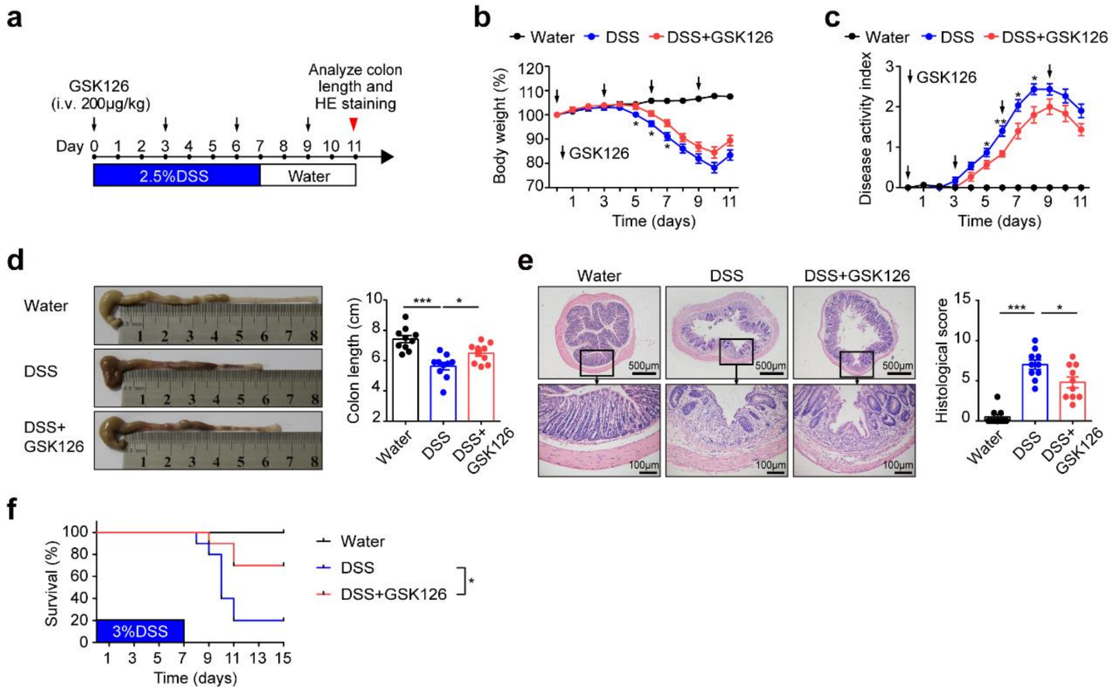

Supplementary Figure 2. Inhibition of EZH2 activity with GSK126 alleviates DSS-induced acute colitis. (**a**) Protocol for DSS-induced acute colitis in C57BL/6 mice and GSK126 administration. (**b**, **c**) Body weight (**b**) and disease activity index (**c**) of mice that received regular drinking water alone or 2.5% DSS or 2.5% DSS combined with GSK126 injection (n=10 per group). Asterisk indicates DSS versus DSS+GSK126. (**d**, **e**) Colon length (**d**), representative H&E staining of histological sections of the distal colon and corresponding histological scores (**e**) at day 11 after DSS administration (n = 10 per group). (**f**) Survival curves from indicated treatment cohorts (n = 10 per group). Data are representative of three independent experiments. *$P < 0.05$ by log-rank test. (**b-e**) Data are representative of three independent experiments. The statistical significance of differences was determined by two-way analysis of variance with Bonferroni post-test (**b, c**) and one-way analysis of variance followed by Bonferroni post-test (**d, e**). *$P < 0.05$, **$P < 0.01$, ***$P < 0.001$. Results are presented as the means ± SEM. Source data are provided as a Source Data file.

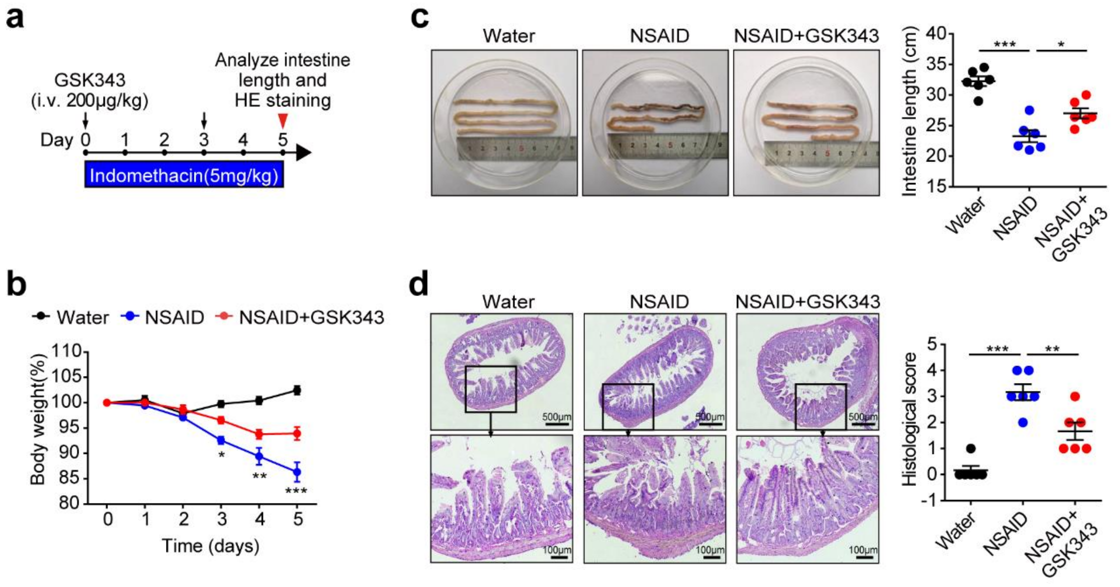

Supplementary Figure 3. Inhibition of EZH2 activity reduced the severity of indomethacin-induced enteropathy. (**a**) The flow diagram of indomethacin-induced acute enteropathy and GSK343 administration. (**b**) Body weight of mice that received regular drinking water alone ("Water" group; Black dotted line) or 5mg/kg indomethacin ("NSAID" group; Blue dotted line) or 5mg/kg indomethacin combined with GSK343 treatment ("NSAID+GSK343" group; Red dotted line) (n = 6 per group). Asterisk indicates "NSAID" group versus "NSAID+GSK343" group. (**c, d**) Intestine length (**c**), representative H&E staining of small intestine sections and corresponding histological scores (**d**) at day 5 after indomethacin administration (n = 6 per group). (**b-d**) Data are representative of three independent experiments. The statistical significance of differences was determined by two-way analysis of variance with Bonferroni post-test (**b**) and one-way analysis of variance followed by Bonferroni post-test (**c, d**). **P* < 0.05, ***P* < 0.01, ****P* < 0.001. Error bars indicate means ± SEM. Source data are provided as a Source Data file.

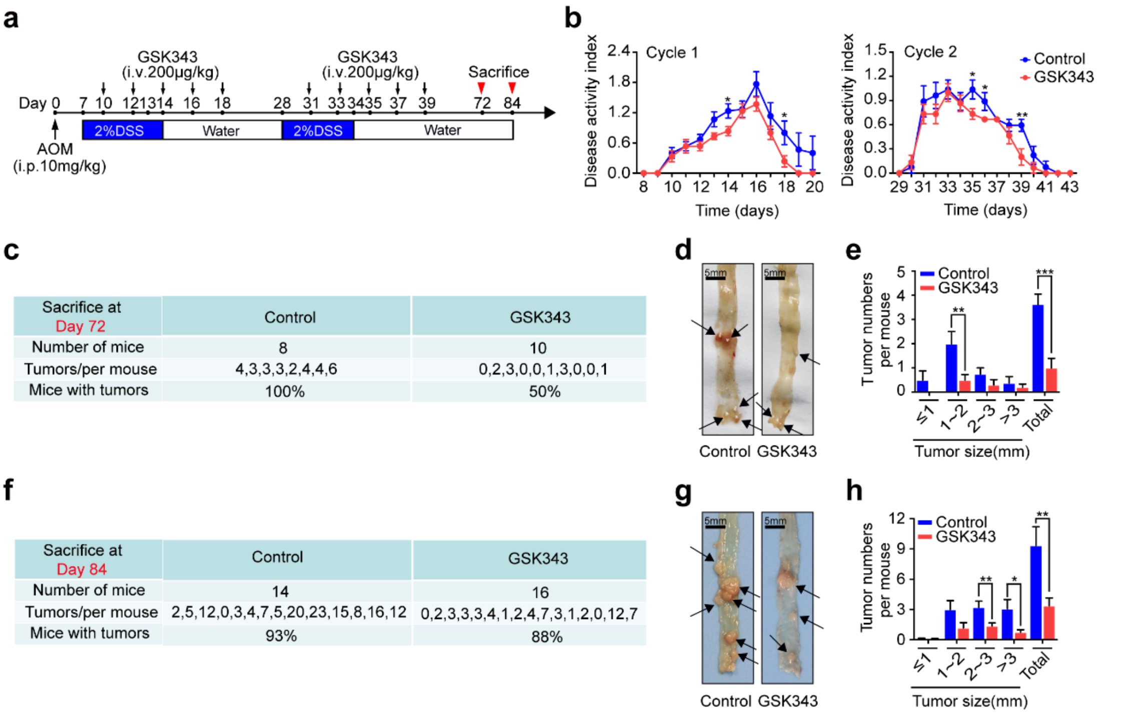

Sacrifice at Day 72	Control	GSK343
Number of mice	8	10
Tumors/per mouse	4,3,3,3,2,4,4,6	0,2,3,0,0,1,3,0,0,1
Mice with tumors	100%	50%

Sacrifice at Day 84	Control	GSK343
Number of mice	14	16
Tumors/per mouse	2,5,12,0,3,4,7,5,20,23,15,8,16,12	0,2,3,3,3,4,1,2,4,7,3,1,2,0,12,7
Mice with tumors	93%	88%

Supplementary Figure 4. Therapeutic inhibition of EZH2 activity during colitis reduces colitis-associated colorectal cancer (CAC). C57BL/6 mice were given a single intraperitoneal injection of azoxymethane (AOM) followed by 2 cycles of 2% DSS. During each cycle, mice were intravenous injected with either vehicle (control) or GSK343 every other day for 5 times when more than half of mice presented colitis symptoms. (**a**) Induction procedure for AOM/DSS-induced CAC in C57BL/6 mice and GSK343 administration. (**b**) Disease activity index during each treatment cycle (n = 9-10 for control; n=10 for GSK343 group). (**c-e**) Summarized results (**c**), representative images of colorectal tumors (**d**), number and size distribution of colorectal tumors (**e**) at day 72 in AOM/DSS-induced mice treated with or without GSK343 (n = 8 for control; n = 10 for GSK343). (**f-h**) Summarized results (**f**), representative images of colorectal tumors (**g**), number and size distribution of colorectal tumors (**h**) at day 84 of the indicated groups (n=14 for control; n =16 for the GSK343 group). (**d, g**) Arrows indicate colorectal tumors. Throughout, data are representative of three independent experiments. The statistical significance of differences was determined by two-way analysis of variance followed by Bonferroni post-test (**b**) and unpaired two-tailed Student's *t*-test (**e, h**). *P < 0.05, **P < 0.01, ***P < 0.001. Error bars indicate means ± SEM. Source data are provided as a Source Data file.

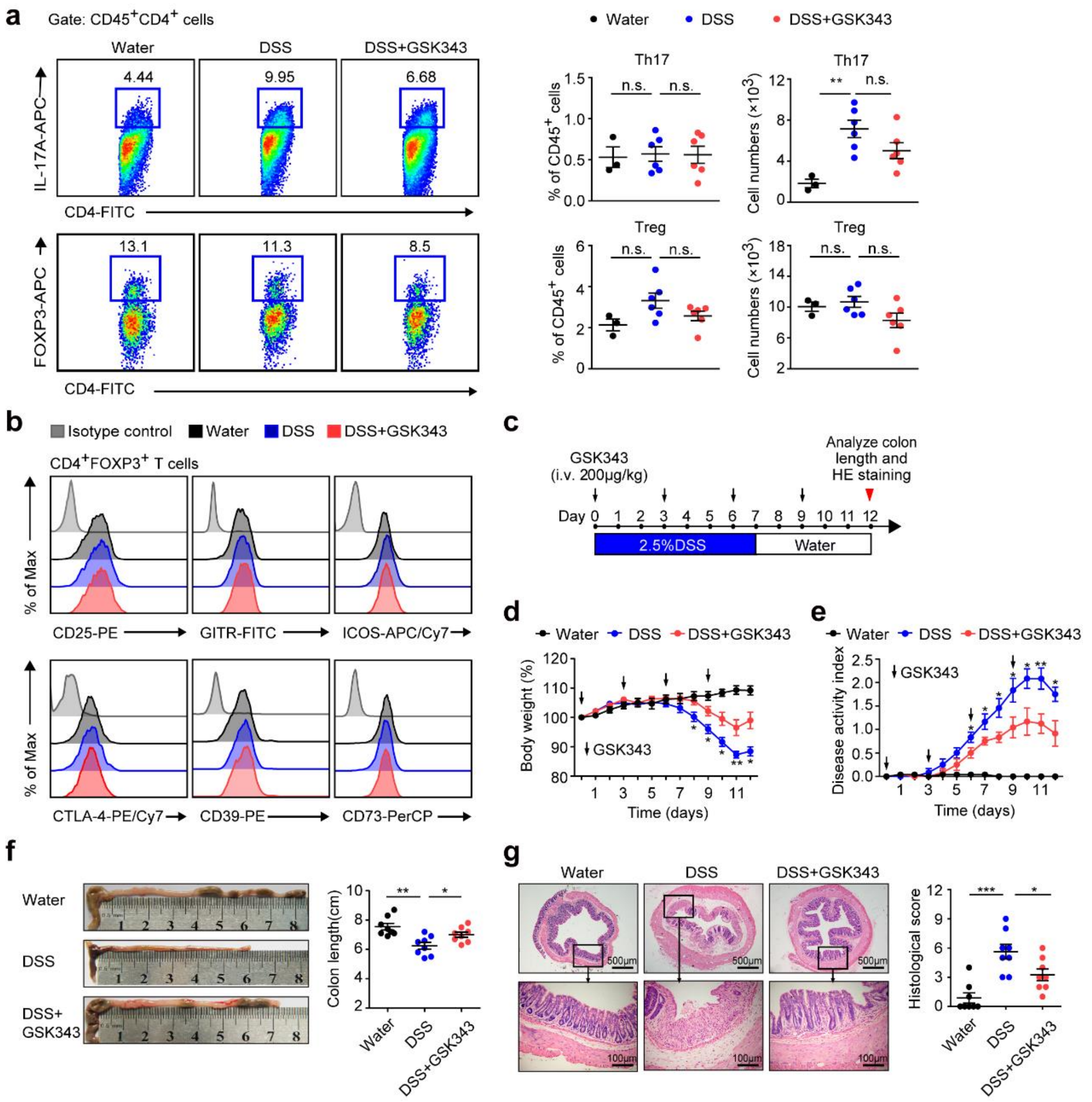

Supplementary Figure 5. Pharmacological inhibition of EZH2 activity attenuates DSS-induced acute colitis independent of influence on adaptive immunity. (**a, b**) C57BL/6 mice were exposed to either 2.5% DSS-containing or regular drinking water alone for 5 days. DSS-treated mice were intravenous injection with either vehicle or GSK343 at day 0, 3. (**a**) At day 5, representative staining, frequencies and absolute numbers of IL-17A^{+} Th17 cells and Foxp3^{+} Treg cells in CD45^{+} cells from colonic lamina propria (cLP) (n = 3, Water; n = 6 for both DSS and DSS+GSK343). Dot plots are gated on CD45^{+} CD4^{+} cells. Numbers adjacent to outlined areas indicate the percentage of cells for the indicated markers. (**b**) Five days post DSS administration, the expression of cell-surface markers in Treg (CD4^{+} Foxp3^{+}) cells isolated from cLP was determined by flow cytometry (n = 3-5 for Water; n = 5-7 for both DSS and DSS+GSK343). (**c**) Methods for DSS-induced colitis in NOD/SCID mice and GSK343 administration. (**d, e**) Changes

in body weight (**d**) and disease activity index (**e**) of mice that received regular drinking water alone or 2.5% DSS or 2.5% DSS combined with GSK343 injection (n =8 per group). (**f, g**) Representative images of colons and colon length (**f**), representative H&E staining of distal colon sections and corresponding histological scores (**g**) at day 12 after DSS exposure (n=8 per group). Throughout, data are representative of three independent experiments and are expressed as the mean ± SEM. The statistical significance of differences was determined by one-way analysis of variance followed by Bonferroni post-test (**a, f, g**) and two-way analysis of variance followed by Bonferroni post-test (**d, e**). *$P < 0.05$, **$P < 0.01$, ***$P < 0.001$, n.s. = not significant. Source data are provided as a Source Data file.

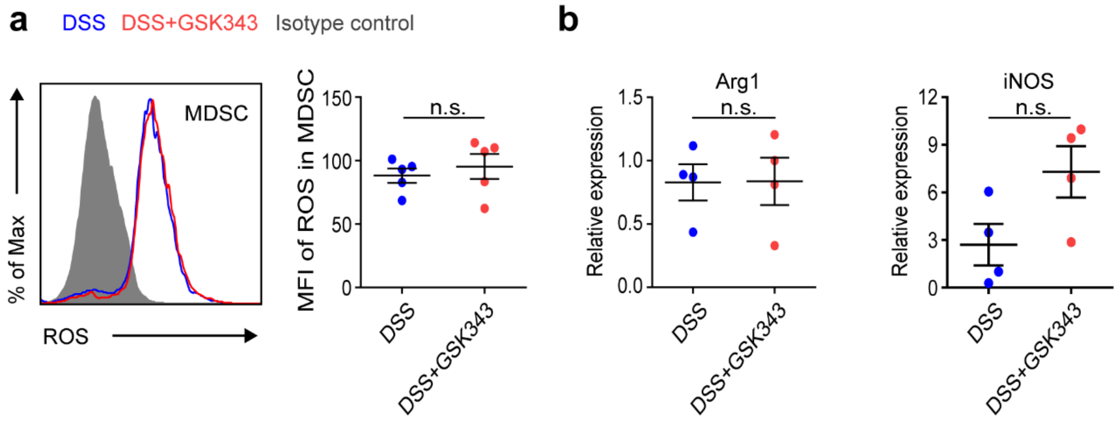

Supplementary Figure 6. Chemical inhibition of EZH2 activity has no obvious effect on MDSCs function during DSS-induced acute colitis. C57BL/6 mice were exposed to 2.5% DSS in the drinking water, tail vein-injected with vehicle or GSK343 at day 0, 3. At day 5, (**a**) histograms and MFI quantification of ROS in colonic MDSCs (CD11b$^+$ Gr-1$^+$) isolated from DSS-exposed mice that were treated with vehicle (blue) or GSK343 (red) (n=5 per group). (**b**) The expression of Arg-1 and iNOS in colonic MDSCs (CD11b$^+$ Gr-1$^+$) from cLP was examined by real-time PCR (n = 4 per group). All experiments were performed at least three times and the data are expressed as means ± SEM. n.s. = not significant (as determined by unpaired two-tailed Student's *t*-test). Source data are provided as a Source Data file.

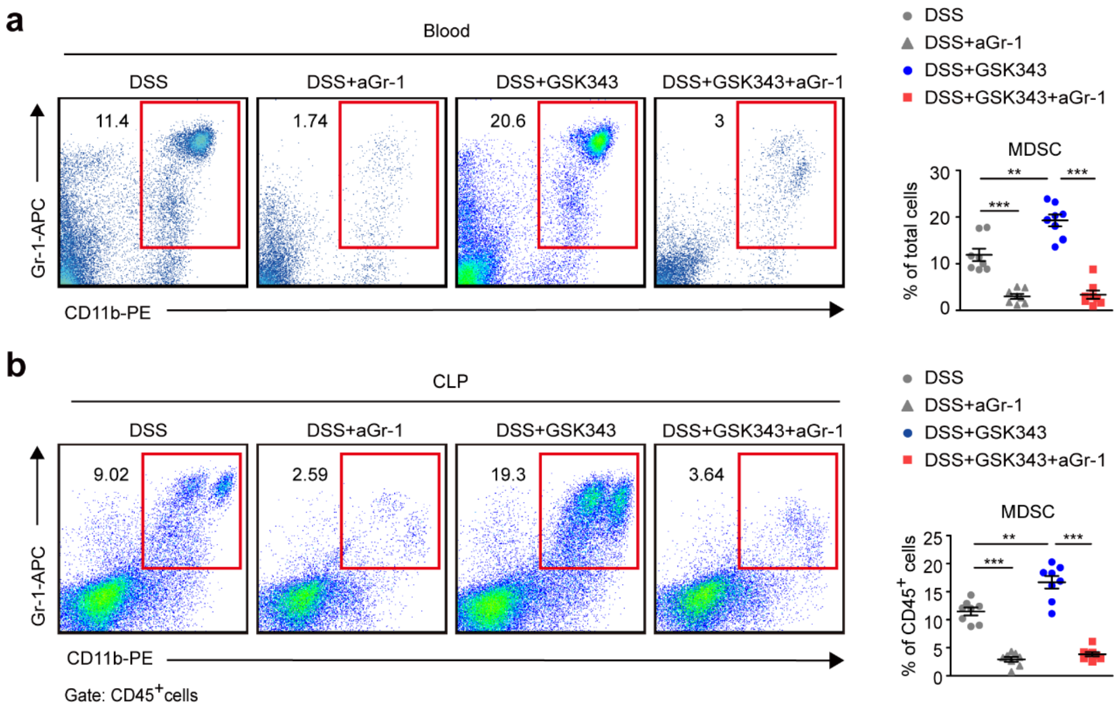

Supplementary Figure 7. Injection of the monoclonal antibody to Gr-1 (aGr-1) efficiently depletes MDSCs in the peripheral blood and cLP. C57BL/6 mice were challenged with 2.5% DSS in the drinking water and intravenously injected with or without GSK343 at day 0, 3. Anti-Gr-1 antibody or isotype control was then administered intraperitoneally into the indicated mice at day3. (**a**) Representative flow cytometry dot plots and relative proportion of CD11b^{+} Gr-1^{+} MDSCs in the peripheral blood at day 4 after DSS treatment (n = 8 per group). (**b**) Representative flow cytometry dot plots and relative proportion of CD11b^{+} Gr-1^{+} MDSCs in CD45^{+} cells among cLP at day 5 after DSS treatment (n = 8 per group). Throughout, numbers adjacent to outlined areas indicate percentage of CD11b^{+} Gr-1^{+} cells in each group. Data are representative of three independent experiments. ***P* < 0.01, ****P* < 0.001 (as determined by one-way analysis of variance with Bonferroni post-test). Error bars indicate means ± SEM. Source data are provided as a Source Data file.

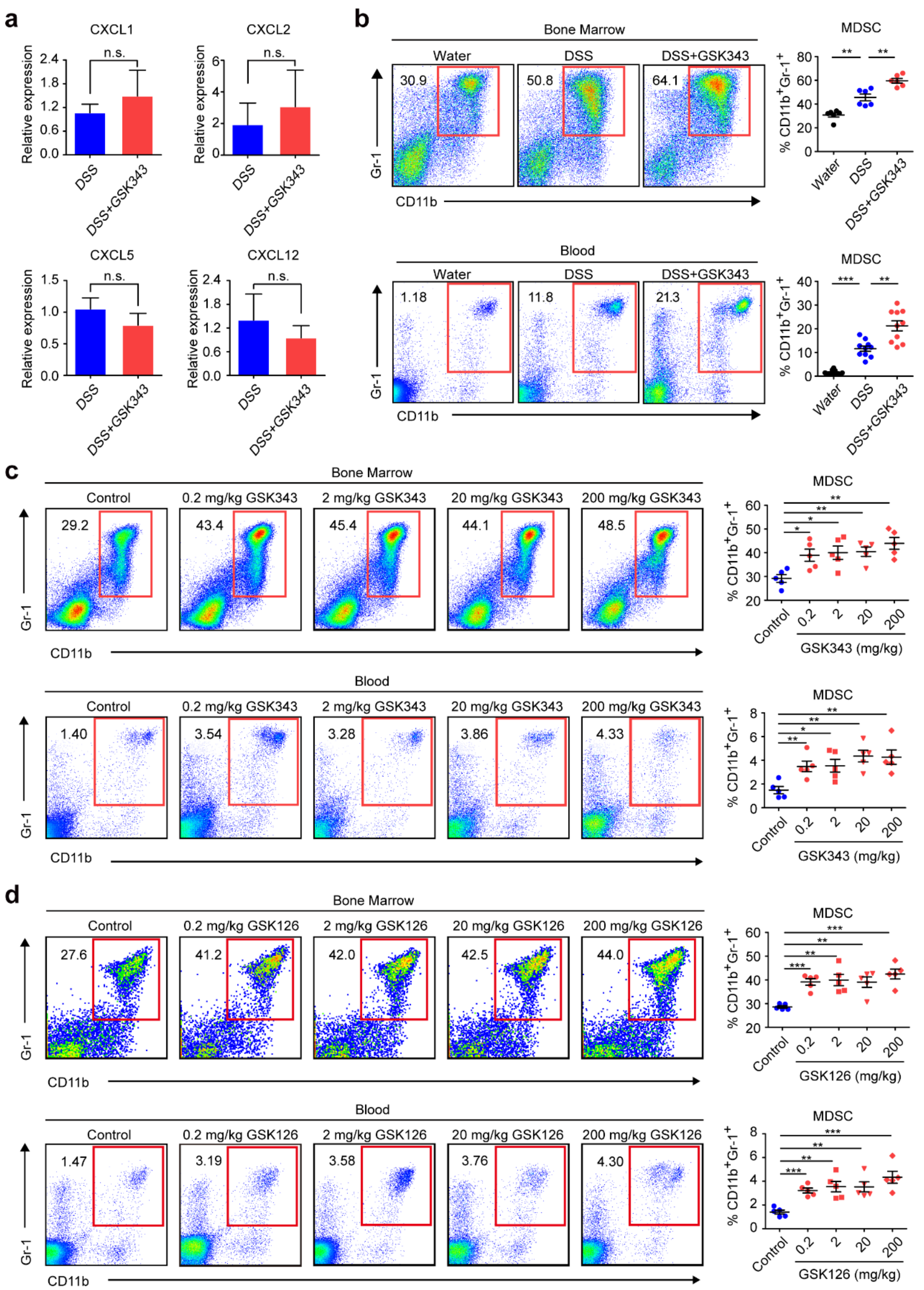

Supplementary Figure 8. Increased colonic MDSCs after EZH2 inhibition may be due to increased MDSCs generation in the bone marrow. (**a, b**) C57BL/6 mice were administered drinking water supplemented with 2.5% DSS, and intravenously injected with vehicle control or

GSK343 at day 0, 3. (**a**) At day5, the expression of MDSC-related chemokines within distal colonic tissues were detected by quantitative real-time PCR analysis (n= 4 per group). (**b**) Representative flow cytometry plots and frequency of $CD11b^{+}Gr\text{-}1^{+}$ MDSCs in the bone marrow (n= 6 per group) and peripheral blood (n= 10 per group) at day 4 after DSS induction. (**c**) C57BL/6 mice were injected intravenously at day 0 and 3 with vehicle or with different doses of GSK343 as indicated. At day 4, the representative dot plots and frequencies of MDSCs ($CD11b^{+}$ $Gr\text{-}1^{+}$) in the bone marrow and peripheral blood (n= 5 per group) were determined by flow cytometry. (**d**) Vehicle or different doses of GSK126 were injected intravenously into C57BL/6 mice at day 0 and 3. At day 4, the representative dot plots and frequencies of MDSCs ($CD11b^{+}$ $Gr\text{-}1^{+}$) in the bone marrow and peripheral blood (n= 5 per group) were determined by flow cytometry. Throughout, data are representative of three independent experiments. *$P < 0.05$, **$P < 0.01$, ***$P < 0.001$, n.s.= not significant (as determined by one-way analysis of variance followed by Bonferroni post-test). Results are presented as the means ± SEM. Source data are provided as a Source Data file.

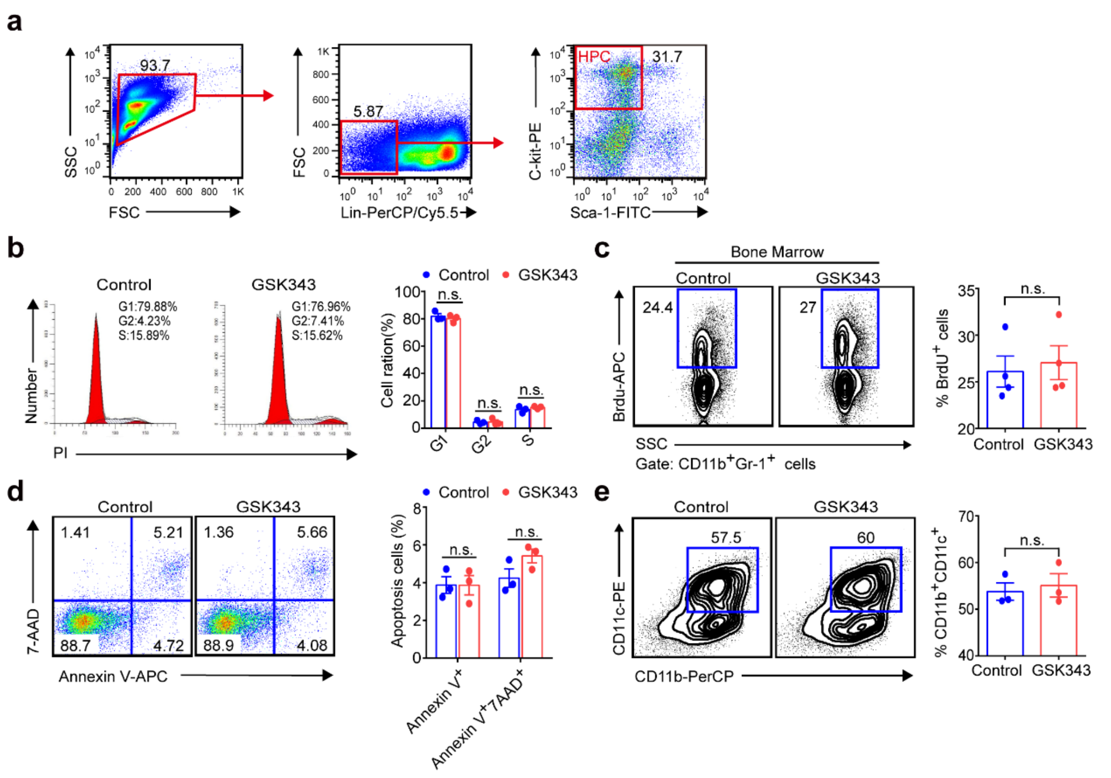

Supplementary Figure 9. EZH2 inhibitor did not apparently affect the proliferation, apoptosis and differentiation of MDSCs. (**a**) Gating strategy for HPC (Lin$^-$Sca-1$^-$C-kit$^+$). (**b**) Sort-purified MDSCs from bone marrow of C57BL/6 mice were cultured with vehicle (control) or GSK343 for 3 days, the cell cycle distribution was then examined by flow cytometry (n=3 per group). (**c**) C57BL/6 mice were injected intravenously with vehicle or GSK343 at day 0 and day 3. At day 4, BrdU incorporation by MDSCs in the bone marrow was analyzed by flow cytometry (n=4 per group). Dot plots are gated on CD11b$^+$Gr-1$^+$ MDSCs. Numbers adjacent to outlined areas indicate the percentage of BrdU$^+$ cells. (**d**) Sort-purified MDSCs from bone marrow were treated with vehicle or GSK343 for 3 days, representative staining and frequencies of AnnexinV$^+$7AAD$^-$（early apoptotic cells）and AnnexinV$^+$7AAD$^+$（late apoptotic cells）cells were assessed by flow cytometry (n=3 per group). Numbers in plots indicate the percentage of cells for the indicated markers. (**e**) *In vitro* incubation of bone marrow-derived MDSCs with GM-CSF (10ng/ml) and vehicle (control) or GSK343 for 5 days. Flow cytometry of the percentage of CD11b$^+$ CD11c$^+$ DC (n=3 per group). (**b-e**) Data are representative of three independent experiments. n.s.= not significant (as determined by unpaired two-tailed Student’s *t*-test). Error bars indicate means ± SEM. Source data are provided as a Source Data file.

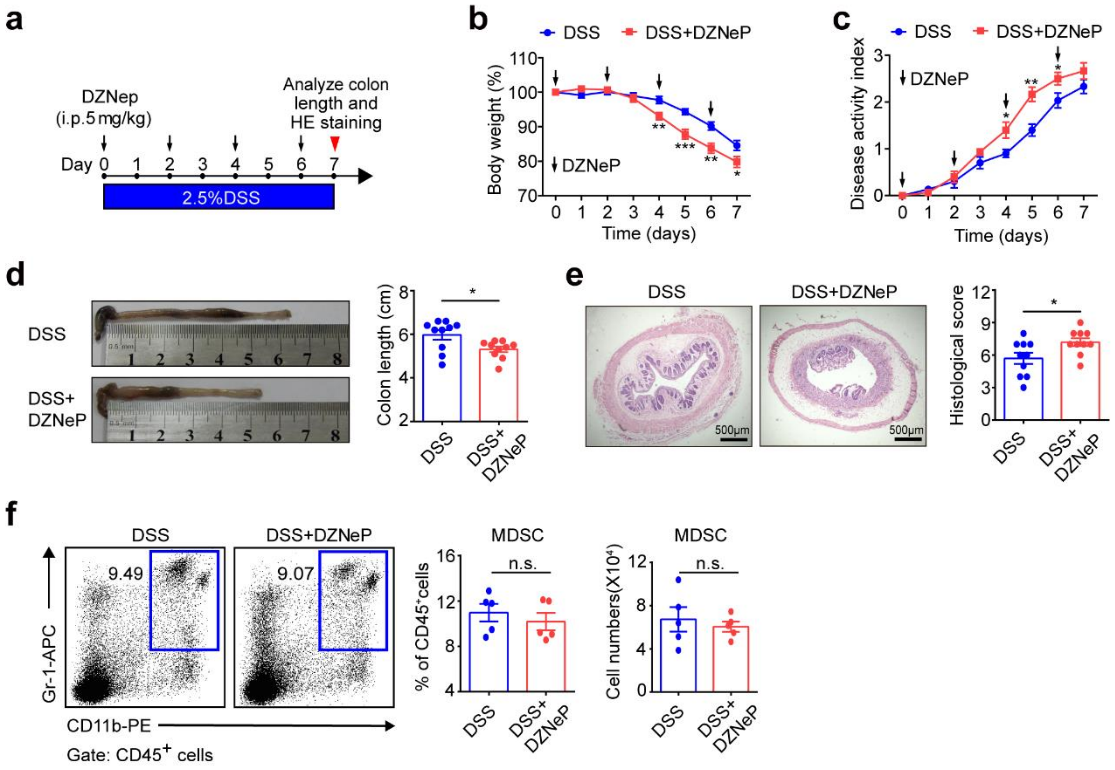

Supplementary Figure 10. Inhibition of EZH2 with DZNeP aggravates colitis. (**a**) Protocol for DSS colitis in C57BL/6 mice and DZNeP administration. (**b**, **c**) Body weight (**b**) and disease activity index (**c**) of mice that received 2.5% DSS or 2.5% DSS combined with DZNeP injection (n=10 per group). Asterisk indicates DSS versus DSS+ DZNeP. (**d**, **e**) Colon length (**d**), representative H&E staining of histological sections of the distal colon and corresponding histological scores (**e**) at day 7 after DSS administration (n = 10 per group). (**f**) At day 5, the representative staining, percentage and absolute number of MDSCs ($CD11b^+$ $Gr\text{-}1^+$) in $CD45^+$ cells of the cLP were determined by flow cytometry (n = 5 per group). Dot plots are gated on $CD45^+$ cells. Numbers adjacent to the outlined areas indicate the percentage of the gated population in each group. Data are representative of three independent experiments. *$P < 0.05$ by log-rank test. (**b-f**) Data are representative of three independent experiments. The statistical significance of differences was determined by two-way analysis of variance with Bonferroni post-test (**b, c**) and unpaired two-tailed Student's *t*-test (**d-f**). *$P < 0.05$, **$P < 0.01$, ***$P < 0.001$, n.s. = not significant. Results are presented as the means ± SEM. Source data are provided as a Source Data file.

Supplementary Table 1. Primer sequences for real-time PCR analyses.

Arg-1	Forward	CTCCAAGCCAAAGTCCTTAGAG
	Reverse	AGGAGCTGTCATTAGGGACATC
iNOS	Forward	CCAAGCCCTCACCTACTTCC
	Reverse	CTCTGAGGGCTGACACAAGG
CXCL1	Forward	GCCTCTAACCAGTTCCAGCA
	Reverse	TTGAGGTGAATCCCAGCCAT
CXCL2	Forward	GGCAAGGCTAACTGACCTGG
	Reverse	CTCAGACAGCGAGGCACATC
CXCL5	Forward	GCCCTACGGTGGAAGTCATA
	Reverse	GAACACTGGCCGTTCTTTCC
CXCL12	Forward	GGTGCTCAAACCTGACGGTA
	Reverse	GGCAGCTCCTCTTTGGCTTA
GAPDH	Forward	CCCATGGCAAATTCCATGGCA
	Reverse	ACGGCAGGTCAGGTCCACC
