# Supplementary material for: Targeting EZH2 histone methyltransferase activity alleviates experimental intestinal inflammation
Source: Nat Commun. 2019 Jun 3;10:2427. doi: 10.1038/s41467-019-10176-2 (PMC6547712; doi:10.1038/s41467-019-10176-2)
Supplement: Supplementary file 3 — Reporting Summary [file 41467_2019_10176_MOESM3_ESM.pdf]

nature research

Corresponding author(s): Bo Zhu

Last updated by author(s): Mar 26, 2019

Reporting Summary

Nature Research wishes to improve the reproducibility of the work that we publish. This form provides structure for consistency and transparency in reporting. For further information on Nature Research policies, see Authors & Referees and the Editorial Policy Checklist.

Statistics

For all statistical analyses, confirm that the following items are present in the figure legend, table legend, main text, or Methods section.

n/a	Confirmed	
☐	☒	The exact sample size (*n*) for each experimental group/condition, given as a discrete number and unit of measurement
☒	☐	A statement on whether measurements were taken from distinct samples or whether the same sample was measured repeatedly
☐	☒	The statistical test(s) used AND whether they are one- or two-sided<br>*Only common tests should be described solely by name; describe more complex techniques in the Methods section.*
☒	☐	A description of all covariates tested
☒	☐	A description of any assumptions or corrections, such as tests of normality and adjustment for multiple comparisons
☐	☒	A full description of the statistical parameters including central tendency (e.g. means) or other basic estimates (e.g. regression coefficient) AND variation (e.g. standard deviation) or associated estimates of uncertainty (e.g. confidence intervals)
☐	☒	For null hypothesis testing, the test statistic (e.g. *F*, *t*, *r*) with confidence intervals, effect sizes, degrees of freedom and *P* value noted<br>*Give P values as exact values whenever suitable.*
☒	☐	For Bayesian analysis, information on the choice of priors and Markov chain Monte Carlo settings
☒	☐	For hierarchical and complex designs, identification of the appropriate level for tests and full reporting of outcomes
☐	☒	Estimates of effect sizes (e.g. Cohen's *d*, Pearson's *r*), indicating how they were calculated

Our web collection on statistics for biologists contains articles on many of the points above.

Software and code

Policy information about availability of computer code

Data collection	No software was used
Data analysis	Flow cytometry data were analyzed using FlowJo software. Graphs were prepared and analysized using Graphpad Prism(version 5.01) software. RT-PCR was analyzed using the 2−ΔΔCt quantification method. For immunofluorescence staining, the number of positive cells per field of view under 800× magnification was counted, and data were collected from five randomly selected fields. The RNA-seq libraries for RNA samples were constructed according to the standard Illumina protocols and sequenced on an Illumina HiSeq 2000 sequencer. The abundance of transcripts was calculated and normalized in fragments per kilobase of transcript per million mapped reads (FPKM) from the raw RNA-seq data. Kyoto Encyclopedia of Genes and Genomes (KEGG) was used to interpret gene expression profile.

For manuscripts utilizing custom algorithms or software that are central to the research but not yet described in published literature, software must be made available to editors/reviewers. We strongly encourage code deposition in a community repository (e.g. GitHub). See the Nature Research guidelines for submitting code & software for further information.

Data

Policy information about availability of data

All manuscripts must include a data availability statement. This statement should provide the following information, where applicable:

- Accession codes, unique identifiers, or web links for publicly available datasets
- A list of figures that have associated raw data
- A description of any restrictions on data availability

The data that support the findings of this study are available from the corresponding author upon reasonable request.

Field-specific reporting

Please select the one below that is the best fit for your research. If you are not sure, read the appropriate sections before making your selection.

☒ Life sciences ☐ Behavioural & social sciences ☐ Ecological, evolutionary & environmental sciences

For a reference copy of the document with all sections, see nature.com/documents/nr-reporting-summary-flat.pdf

Life sciences study design

All studies must disclose on these points even when the disclosure is negative.

Sample size	For each individual experiments, the sample size was not predetermined before initial experiment. Normally 3 or up to 18 biological repeats were included based on experience or the sample availability. After the initial experiment, power analyses were performed to decide the final sample size.
Data exclusions	No data exclusions were taken for this manuscript.
Replication	Each experiment was repeated at least three times as described in Figure legends. Experimental findings were reliably reproduced between these experiments.
Randomization	Mice used in this experiment were randomly assigned to different groups.
Blinding	N/A

Reporting for specific materials, systems and methods

We require information from authors about some types of materials, experimental systems and methods used in many studies. Here, indicate whether each material, system or method listed is relevant to your study. If you are not sure if a list item applies to your research, read the appropriate section before selecting a response.

Materials & experimental systems

n/a	Involved in the study	
☐	☒	Antibodies
☒	☐	Eukaryotic cell lines
☒	☐	Palaeontology
☐	☒	Animals and other organisms
☒	☐	Human research participants
☒	☐	Clinical data

Methods

n/a	Involved in the study	
☒	☐	ChIP-seq
☐	☒	Flow cytometry
☒	☐	MRI-based neuroimaging

Antibodies

Antibodies used	Fluorophore- or biotin-conjugated antibodies specific for mouse cell-surface antigens and cytokines were used as follows: anti-CD45 (FITC, BioLegend, clone 30-F11, catalog 103108), anti-CD11b (PE, Biolegend, clone M1/70, catalog 101208; PerCP, Biolegend, clone M1/70, catalog 101230), anti-CD11c (PE, BD Pharmingen™, clone HL3, catalog 557401), anti-F4/80 (APC, BioLegend, clone BM8, catalog 123116), MHC class II (APC, BioLegend, clone M5/114.1, catalog 107614), anti-Gr-1 (APC, BioLegend, clone RB6-8C5, catalog 108412), anti-Siglec-F (PE, BD Pharmingen™, clone E50-2440, catalog 562068), anti-CD4 (FITC, BioLegend, clone GK1.5, catalog 100406 /Pacific Blue, BioLegend, clone GK1.5, catalog 100428), anti-IL-17A (APC, eBioscience, clone eBio17B7, catalog 17-7177-81), anti-Foxp3 (APC, eBioscience, clone FJK-16s, catalog 171-5773-82), anti-CD25 (PE, BioLegend, clone PC61, catalog 102008), anti-GITR (FITC, BioLegend, clone DTA-1, catalog 126308), anti-ICOS (APC/Cy7, Biolegend, clone C398.4A, catalog 313529), anti-CTLA-4 (PE/Cy7, BioLegend, clone BNI3, catalog 369614), anti-CD39 (PE, BioLegend, clone Duha59, catalog 143803), anti-CD73 (PerCP/Cy5.5, BioLegend, clone TY/11.8, catalog 127214), anti-Lineage (PerCP/Cy5.5, BD Pharmingen™, clone AA4.1, catalog 561317), DCFH-DA (FITC, Beyotime, catalog S0033), PI (Biolegend, catalog 421301) 7-AAD (Biolegend, catalog 420403), Annexin V (APC, Biolegend, catalog 640941), anti-Sca-1 (FITC, BioLegend, clone D7, catalog 108106), anti-C-kit (PE, BioLegend, clone 2B8, catalog 105808), BrdU Flow Kit (APC, BD Pharmingen™, catalog 559619).
Validation	Validation available on the manufacturer's websites

Animals and other organisms

Policy information about studies involving animals; ARRIVE guidelines recommended for reporting animal research

Laboratory animals	C57BL/6, non-obese diabetes/severe combined immunodeficiency (NOD/SCID) mice were purchased from the Chinese Academy

Laboratory animals	of Medical Sciences (Beijing, China). All mice were kept in laminar flow cabinets under a specific pathogen-free environment. Female animals were used for all studies and were 6-8 weeks of age at the start of the experiments.
Wild animals	The study did not involve wild animals
Field-collected samples	The study did not involve samples from the field
Ethics oversight	Mouse care and use were approved by the Third Military Medical University Institutional Animal Care and Use Committee.

Note that full information on the approval of the study protocol must also be provided in the manuscript.

Flow Cytometry

Plots

Confirm that:

- [x] The axis labels state the marker and fluorochrome used (e.g. CD4-FITC).
- [x] The axis scales are clearly visible. Include numbers along axes only for bottom left plot of group (a 'group' is an analysis of identical markers).
- [x] All plots are contour plots with outliers or pseudocolor plots.
- [x] A numerical value for number of cells or percentage (with statistics) is provided.

Methodology

Sample preparation	For cell-surface antigen staining, cells were pre-incubated with Fc Receptors Blocking Reagent (Miltenyi Biotec) for 15min at 4°C before being stained with antibodies. Fixable Viability Dye eFluor® (eBioscience) was added to exclude dead cells. The following mouse antibodies were used for staining: CD45 (Biolegend), CD11b (Biolegend), CD11c (BD Pharmingen™), F4/80 (Biolegend), Gr-1 (Biolegend), Siglec-F (BD Pharmingen™), CD4 (Biolegend), IL-17A (eBioscience), Foxp3 (eBioscience), CD25 (Biolegend), GITR (Biolegend), ICOS (Biolegend), CTLA-4 (Biolegend), CD39 (Biolegend), CD73 (Biolegend), Lineage (BD Pharmingen™), DCFH-DA (Beyotime), PI (Beyotime), Annexin V (Biolegend), 7-AAD (Biolegend). Incorporation of BrdU was detected with an APC BrdU Flow Kit (BD Pharmingen™) according to the manufacturer’s protocol.
Instrument	Flow cytometry and cell sorting was performed using a BD FACSCalibur flow cytometer and a BD FACSAriaTM II cell sorter, respectively.
Software	Data were analyzed using the FlowJo software.
Cell population abundance	The purity for all populations was >95%.
Gating strategy	Gating strategy: after excluding doublets or larger aggregates, very small events, likely nuclei or debris, were excluded. Positive and negative populations were defined by using fluorescence minus one controls, or staining cells purified from mice lacking the detected protein or appropriate positive and negative controls.

- [x] Tick this box to confirm that a figure exemplifying the gating strategy is provided in the Supplementary Information.
